# Effects of Microgravity on Early Embryonic Development and Embryonic Stem Cell Differentiation: Phenotypic Characterization and Potential Mechanisms

**DOI:** 10.3389/fcell.2021.797167

**Published:** 2021-12-02

**Authors:** Feng Li, Ying Ye, Xiaohua Lei, Wensheng Zhang

**Affiliations:** ^1^ Department of Urinary Surgery, The First Affiliated Hospital of Soochow University, Suzhou, China; ^2^ Cam-Su Genomic Resource Center, Medical College of Soochow University, Suzhou, China; ^3^ Center for Energy Metabolism and Reproduction, Shenzhen Institutes of Advanced Technology, Chinese Academy of Sciences, Shenzhen, China; ^4^ Department of Physiology, School of Basic Medical Sciences, Binzhou Medical University, Yantai, China

**Keywords:** microgravity, ES cells, differentiation, self-renewal, embryonic developement

## Abstract

With the development of science and technology, mankind’s exploration of outer space has increased tremendously. Settling in outer space or on other planets could help solve the Earth’s resource crisis, but such settlement will first face the problem of reproduction. There are considerable differences between outer space and the Earth’s environment, with the effects of gravity being one of the most significant. Studying the possible effects and underlying mechanisms of microgravity on embryonic stem cell (ESC) differentiation and embryonic development could help provide solutions to healthy living and reproduction in deep space. This article summarizes recent research progress on the effects of microgravity on ESCs and early embryonic development and proposes hypotheses regarding the potential mechanisms. In addition, we discuss the controversies and key questions in the field and indicate directions for future research.

## Introduction

All creatures on Earth are impacted by gravity. When the human body is weightless in space, many physiological functions change, including bone loss, muscle atrophy, decreased cardiovascular capacity, decreased immune function, delayed wound healing, and delayed fracture healing (Lackner and DiZio, 2000; Blaber et al., 2014). With expanding space exploration, humans will inevitably remain in these environments for longer periods of time and may eventually need to reproduce. Therefore, studying the effects of space on human reproduction and development has become a hot topic in space biology research (Blaber et al., 2015; Lei et al., 2019).

Embryonic stem cells (ESCs) are derived from the inner cell mass of preimplantation embryos (Ye et al., 2021). They exhibit indefinite self-renewal *in vitro* and maintain the ability to differentiate into different types of cells in the body, i.e., pluripotency, and are therefore widely used to study reproduction and development in mammals (Lee et al., 2017; Ye et al., 2021). Various studies have shown that ESC self-renewal and pluripotency are controlled by a network of signal transduction pathways, transcriptional factors, and chromatin remodeling complexes (Niwa, 2001; Ye et al., 2021). Studying the effects of microgravity on ESC self-renewal and differentiation provides an important way to reveal the impact of the space environment on human reproduction and development.

In this article, we review recent advances in studies on the effects of microgravity on ESCs and early embryonic development, as well as the potential underlying mechanisms.

## Effects of Microgravity on ESC Maintenance

**FIGURE 1:**
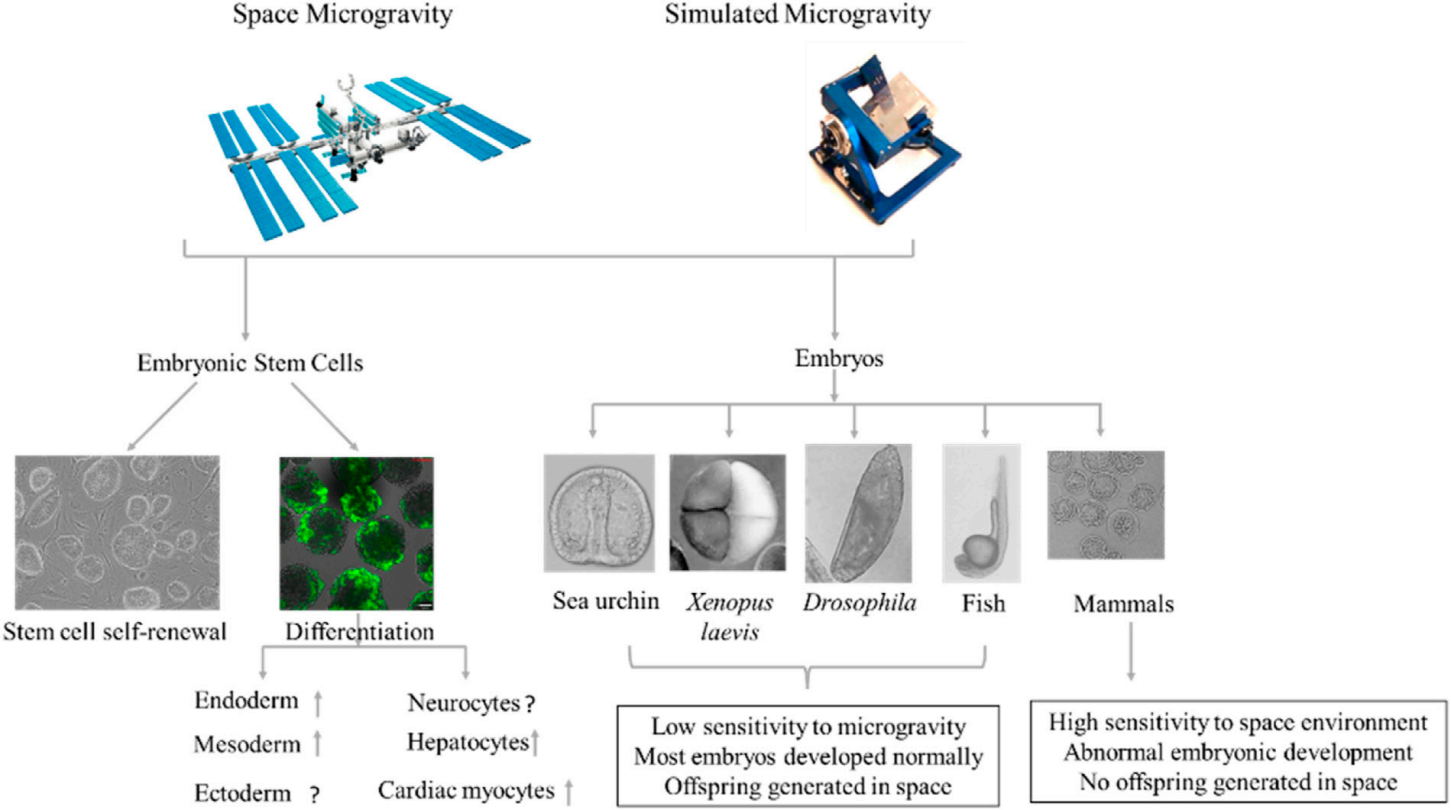
Effects of microgravity on embryonic stem cell (ESC) differentiation and early embryonic development. Microgravity affects ESC self-renewal and differentiation (Left). Space does not appear to adversely affect embryonic development in non-mammalian animals, with some successfully developing and reproducing offspring under microgravity conditions (Ubbels et al., 1989; Vernós et al., 1989; Marthy et al., 1994; Souza et al., 1995; Black et al., 1996; Ijiri, 1998; Schatten et al., 1999). In mammals, however, mice in early-stage pregnancy fail to develop and produce offspring under microgravity, whereas mice in middle- and late-stage pregnancy can successfully produce a viable fetus during space flight and give birth to live pups after landing (Wakayama et al., 2009; Kojima et al., 2000; Jung et al., 2009; Ma et al., 2008; Mishra and Luderer, 2019).

The maintenance of ESCs depends on a variety of synergistic factors (Young, 2011; Ye et al., 2021). Research has shown that mouse ESCs can be maintained without leukemia inhibitory factor (LIF) and retain pluripotency under a simulated microgravity environment (Kawahara et al., 2009). Oct4 is one of the most important transcription factors involved in the maintenance of ESC identity, and changes in its expression can result in ESC differentiation (Shi and Jin, 2010; Ye et al., 2021). Mouse ESCs cultured under a microgravity environment for 15 days using automatic culture equipment aboard a TZ-1 space vehicle show significantly higher cell survival and proliferation as well as *Oct4* expression compared to the ground-based control group (Lei et al., 2018), indicating that microgravity may contribute to the maintenance of ESCs. Similarly, mouse induced pluripotent stem cells (iPSCs) grown under microgravity conditions show greater proliferation ability and newborn cells overgrown in the first 3 days show higher levels of *Oct4* than cells from the ground-based control (Zhou et al., 2019). In contrast, Wang et al. (2011) found that mouse ESC apoptosis increases and adherent cells decrease under a simulated microgravity, resulting in a significant decrease in cell expansion. Acharya et al. (2018) analyzed gene expression in mouse ESCs after exposure to alternating hypergravity and microgravity and detected changes in the expression of genes related to cell cycle and cell proliferation, indicating that gravity affects the proliferation of ESCs. Thus, growing evidence suggests that microgravity can significantly affect the self-renewal of ESCs (Figure 1).

RNA sequencing (RNA-seq) of mouse ESCs carried on the SJ10 recoverable satellite found down-regulation of genes related to DNA repair in the space environment, suggesting a negative impact of space on the maintenance of genomic stability (An et al., 2019). In addition to microgravity, cells exposed to the space environment also experience strong cosmic radiation. Therefore, the impact of the space environment on cells is likely caused by the dual effects of microgravity and radiation.

Reactive oxygen species (ROS) are necessary for DNA repair pathways to maintain genome stability; however, excessive ROS can cause cell damage and apoptosis (Li and Marbán, 2010). Rad9 is an important component of the DNA damage response (DDR) system. Studies have shown that simulated microgravity can promote the formation of ROS, thereby increasing the sensitivity of DNA to excess ROS, leading to significant DNA double-strand breaks in ESCs lacking Rad9 (Li et al., 2015; Ran et al., 2016). In addition, as microgravity incubation time increases, ROS levels return to normal and DNA damage decreases, though not to control levels, indicating that microgravity may be a weak stress for genomic DNA damage, but it aggravates genomic DNA instability in defective ESCs in the DDR system (Li et al., 2015; Ran et al., 2016). However, Lei et al. (2020) reported that simulated microgravity itself has no significant effect on DNA damage in mouse blastocysts, indicating that microgravity alone may not have a significant impact on DNA damage in ESCs. However, microgravity may cooperate with other factors, such as radiation and the DDR system, to regulate DNA damage in coordination (Li et al., 2015; Ran et al., 2016; Lei et al., 2020).

## Effects of Microgravity on ESC Differentiation

ESCs can differentiate into different types of cells in the body. Multiple studies have shown that microgravity can affect the differentiation of ESCs (Lei et al., 2014; Blaber et al., 2015; Jha et al., 2016; Shinde et al., 2016; Lei et al., 2018; Mattei et al., 2018; Oss-Ronen et al., 2020). For example, the expression of mesoderm marker gene Brachyury (T) in embryoid bodies (EBs) is significantly higher under microgravity than under 1G ground conditions (Lei et al., 2018; Oss-Ronen et al., 2020), indicating that microgravity promotes the differentiation of ESCs into mesoderm. A study from NASA (STS-131 mission on the space shuttle Discovery) showed that ESCs differentiate more readily into contractile cardiomyocytes under microgravity conditions (Blaber et al., 2015). Consistently, microarray data analysis found that genes related to heart morphogenesis are up-regulated in ESCs cultured for 3 days under microgravity (Shinde et al., 2016). Furthermore, after transfer to normal gravity, the expression levels of myocardial-specific genes *Tnnt2*, *Rbp4*, *Tnni1*, *Csrp3*, *Nppb*, and *Mybpc3* are suppressed, indicating that simulated microgravity may promote the differentiation of ESCs into myocardium Consolo et al. (2012) (Shinde et al., 2016). optimized the differentiation of ESCs into cardiomyocytes under microgravity conditions and Jha et al. (2016) found that human iPSCs can differentiate into functional cardiomyocytes under simulated microgravity. Microgravity exposure is also reported to activate the expression of *CDC42* through Rap1GDS1, thereby promoting the occurrence of vascular branch morphology (Wang et al., 2017). Mouse ESCs can differentiate into hepatic-like cells under simulated microgravity environments, which show the morphological characteristics of mature hepatocytes and typical expression of hepatic-specific genes and proteins (Wang et al., 2012). In addition, cells exposed to simulated microgravity can also differentiate into liver-like cells when transplanted into mice (Wang et al., 2012). Similarly, Zhang et al. (2013) found that microgravity simulation can promote the efficiency of liver differentiation of mouse ESCs, and these differentiated cells can be successfully transplanted into a recipient liver.

In addition to the increase in mesoderm differentiation, the expression levels of endoderm markers, such as *FoxA2*, *Sox17*, and *CxCr4*, are also significantly up-regulated in differentiated ESCs under microgravity, indicating that microgravity may promote the differentiation of ESCs into endoderm (Oss-Ronen et al., 2020). Consistently, Lei et al. (2014) showed that microgravity promotes the differentiation of mouse ESCs into endoderm by activating the Wnt pathway but inhibits the differentiation of ESCs into neuroectoderm. Furthermore, inhibition of the Wnt/β-catenin pathway by DKK1 down-regulates the expression of T and other mesendoderm marker genes (Lei et al., 2014). Mattei et al. (2018) studied the influence of microgravity on differentiation of human ESCs into neural organoids and found that microgravity biases the differentiation of hESCs towards caudal neural progenitor types. However, whether microgravity influences proliferation and/or differentiation of specific cell types within neural organoids is unclear (Mattei et al., 2018).

In summary, the microgravity environment can affect the differentiation of ESCs into the three germ layers (Figure 1). Multiple research groups have shown that microgravity promotes the differentiation of ESCs into mesoderm, endoderm, and their differentiated cells. However, the differentiation of ESCs into ectoderm is more complicated and needs further research.

## Mechanisms Underlying Effects of Microgravity on ESC Maintenance and Differentiation

Various studies have shown that a network of signaling pathways, transcription factors, microRNAs, and chromatin remodeling complexes control the maintenance and differentiation of ESCs into the three germ layers (Ye et al., 2021). Due to technical and experimental limitations, studies on the effects of microgravity on the maintenance and differentiation of ESCs are mostly descriptive, with in-depth mechanistic studies remaining scarce.

Cells can perceive the mechanical environment through skeletal tension and integrin-mediated focal adhesion, thereby triggering downstream signals, i.e., mechanical conduction signals (Weng et al., 2016; Kaitsuka and Hakim, 2021). The cytoskeleton is one of the most complex and versatile structures in cells and plays important roles in cell division, force conduction, and intracellular signal conduction (Hohmann and Dehghani, 2019). The cytoskeleton changes during ESC differentiation (Fan et al., 2018). The stiffness of undifferentiated ESCs does not change, but increases significantly during neural differentiation, which is regulated by cytoskeletal structures (Fan et al., 2018). Consistently, the expression levels of cytoskeleton-related proteins, such as Fascin-1, Cofilin-1, and Stathmin-1, are up-regulated during differentiation of human ESCs into neural cells (Chae et al., 2009). Shinde et al. (2016) also identified 19 up-regulated cytoskeleton-related genes in ESC-induced EBs under microgravity conditions. Thus, changes in the cytoskeleton under microgravity may be related to the expression of cytoskeleton-related proteins. β-actin and α-tubulin expression levels are down-regulated in mesenchymal stem cells under simulated microgravity, while microtubules and microfilaments are reorganized, leading to cytoskeletal remodeling Feng et al. (2019) (Chi et al., 2020). reported that 12 long noncoding RNAs (lncRNAs) related to microfilament and tubulin expression are differentially expressed during the mouse pronuclear stage under microgravity, with the target genes involved in cellular processes related to cytoskeleton and protein transport. Thus, microgravity may regulate ESC differentiation by affecting the cytoskeletal structure and expression of cytoskeleton-related genes. Although previous studies have shown a correlation between cytoskeletal changes and ESC characteristics under microgravity conditions (Chae et al., 2009; Shinde et al., 2016; Fan et al., 2018), the molecular mechanism underlying the effects of microgravity on the maintenance and differentiation of ESCs *via* regulation of the cytoskeleton has yet to be studied in-depth.

Microgravity exposure has been shown to change the expression of genes related to ESC differentiation as well as signaling pathway activity Lei et al. (2014) (Lei et al., 2014; An et al., 2019). reported that microgravity regulates the differentiation of ESCs into mesendoderm by affecting WNT signaling pathway activity. In addition to the WNT pathway, other canonical signaling pathways, such as TGFβ/BMP, control mesoderm and endoderm specification of ESCs *via* regulation of pluripotency factor expression (Gordeeva, 2019), suggesting that microgravity may regulate the activities of those pathways to control ESC differentiation. The expression of p21/Cdkn1a increases during spaceflight in osteoprogenitors (Blaber et al., 2013). In addition, p21/Cdkn1a plays a mechano-reversible anti-proliferative role during osteogenesis, and thus may regulate proliferation and differentiation during development in response to both the space environment and mechano-stimulation (Juran et al., 2021). Oct4 may maintain ESC proliferation by regulating the expression of p21/Cdkn1a (Lee et al., 2010). Thus, it would be interesting to study whether p21 regulates the balance between ESC proliferation and differentiation under a microgravity environment. Recently, Lei et al. (2020) found that microgravity changes the methylation of DNA at 6,130 sites in the blastocyst cell genome, indicating that microgravity may regulate gene expression *via* DNA methylation modification and participate in the development of mouse embryos. Functional clustering analysis also indicated that those loci with DNA methylation changes are related to histone modification, chromatin structure, cytoskeletal structure, and RNA metabolism regulation (Lei et al., 2020). Thus, microgravity may also regulate the maintenance and differentiation of ESCs *via* epigenetic modification of genes and chromatin structure. However, in-depth studies are needed to clarify these possibilities.

## Effects of Microgravity on Early Embryonic Development and Underlying Mechanism

Clarifying the effects of microgravity on human embryonic development is difficult due to ethical concerns and technical limitations. However, given the similarities between mouse and human embryonic development, mice can be a useful surrogate tool for studying the effects of microgravity on human embryonic development. Mice exposed to the space environment at the early stage of pregnancy fail to produce viable offspring, whereas mice exposed to space during the middle and late stages of pregnancy are able to successfully give birth to viable offspring upon their return to Earth (Mishra and Luderer, 2019) (Figure 1). This may be related to the development of poor-quality blastocysts following microgravity exposure (Lei et al., 2020). Multiple studies have shown that mice can complete *in vitro* fertilization under simulated microgravity (Kojima et al., 2000; Wakayama et al., 2009), but preimplantation development of fertilized eggs is detrimentally affected (Kojima et al., 2000; 39,; Ma et al., 2008; Wakayama et al., 2009; Jung et al., 2009). Wakayama et al. (2009) reported that *in vitro* fertilized eggs cultured under simulated microgravity conditions show slower development and fewer trophectoderm cells in the blastocysts compared with 1G controls, but no change in blastocyst polarization. Similarly, the number of fertilized eggs that develop to morula and blastocysts is significantly reduced after 96 h of culture under simulated microgravity (Kojima et al., 2000). Among the 49 two-cell mouse embryos sent into space onboard the Columbia Space Shuttle, none developed (Schenker and Forkheim, 1998). Furthermore, among the 100 four-cell mouse embryos delivered to the SJ-8 orbital module platform, none developed into blastocysts (Ma et al., 2008); Jung et al. (2009) used a rotating cell culture system bioreactor with a high aspect ratio vessel to simulate *in vitro* fertilization and embryonic development of cattle under microgravity conditions and found that *in vitro* fertilization did not occur and 2–8-cell embryos did not develop to the next stage under such conditions. Lei et al. (2020) reported that two-cell-stage mouse embryos taken to space by the SJ-10 recoverable satellite successfully developed into blastocysts, but with compromised blastocyst formation and quality. Thus, microgravity appears to affect the development of embryos before implantation (Schenker and Forkheim, 1998; Kojima et al., 2000; Ma et al., 2008; Jung et al., 2009; Wakayama et al., 2009). The inconsistent results from different groups may be due to different shear stress caused by different simulated microgravity methods or equipment (Xie et al., 2006) or to species-specific reactions of embryos to microgravity.

The molecular mechanism underlying the effects of microgravity on embryonic development remain unclear. Exploratory studies have been carried out by several research groups (Cao et al., 2007; Rizzo et al., 2009; Wang et al., 2009; Lei et al., 2020). Stress-activated protein kinase (SAPK) belongs to the mitogen-activated protein kinase (MAPK) family and is found in stem cells. It can affect the activity of transcription factors when the cell undergoes a stress response (Puscheck et al., 2015). Wang et al. (2009) reported that the effect of microgravity on cell growth at different embryonic stages is achieved *via* phosphorylation of SAPK, and that SAPK inhibitors can significantly reduce embryonic stress caused by microgravity. However, the apoptotic level of embryonic cells is still higher than that under normal gravity, suggesting that in addition to the SAPK pathway, microgravity may have other ways to cause cell death (Wang et al., 2009).

Nitric oxide (NO) plays an important role in the development of embryos before implantation, with a critical concentration required for normal embryonic development (Tranguch et al., 2003). Several studies have shown that microgravity can affect nitric oxide synthase (NOS) and NO production in mammals (Klein-Nulend et al., 2003; Xiong et al., 2003). Bone cells cultured under simulated microgravity show higher NOS activity and greater NO production (Klein-Nulend et al., 2003). Microgravity exposure increases NO as well as inducible nitric oxide synthase (iNOS) and iNOS mRNA expression in rat cardiac myocytes, partially *via* activation of protein kinase C (Xiong et al., 2003). Cao et al. (2007) found higher NO content and NOS activity in culture medium under microgravity conditions, potentially resulting in retardation of mouse embryonic development and cell apoptosis. Therefore, the microgravity environment may affect embryonic development *via* regulation of NO expression.

Oxidative stress is involved in many embryonic developmental processes. Oxidative stress induced by excessive ROS or insufficient antioxidant protection can detrimentally affect embryonic development Rizzo et al. (2009) (Lin and Wang, 2020). found that both glutathione content and antioxidant enzyme activity in *Xenopus* embryos increase under microgravity conditions, and that the glutathione system is an important mechanism against oxidative stress. Thus, these results suggest that microgravity can cause oxidative stress and affect the development of mouse preimplantation embryos.

**FIGURE 2:**
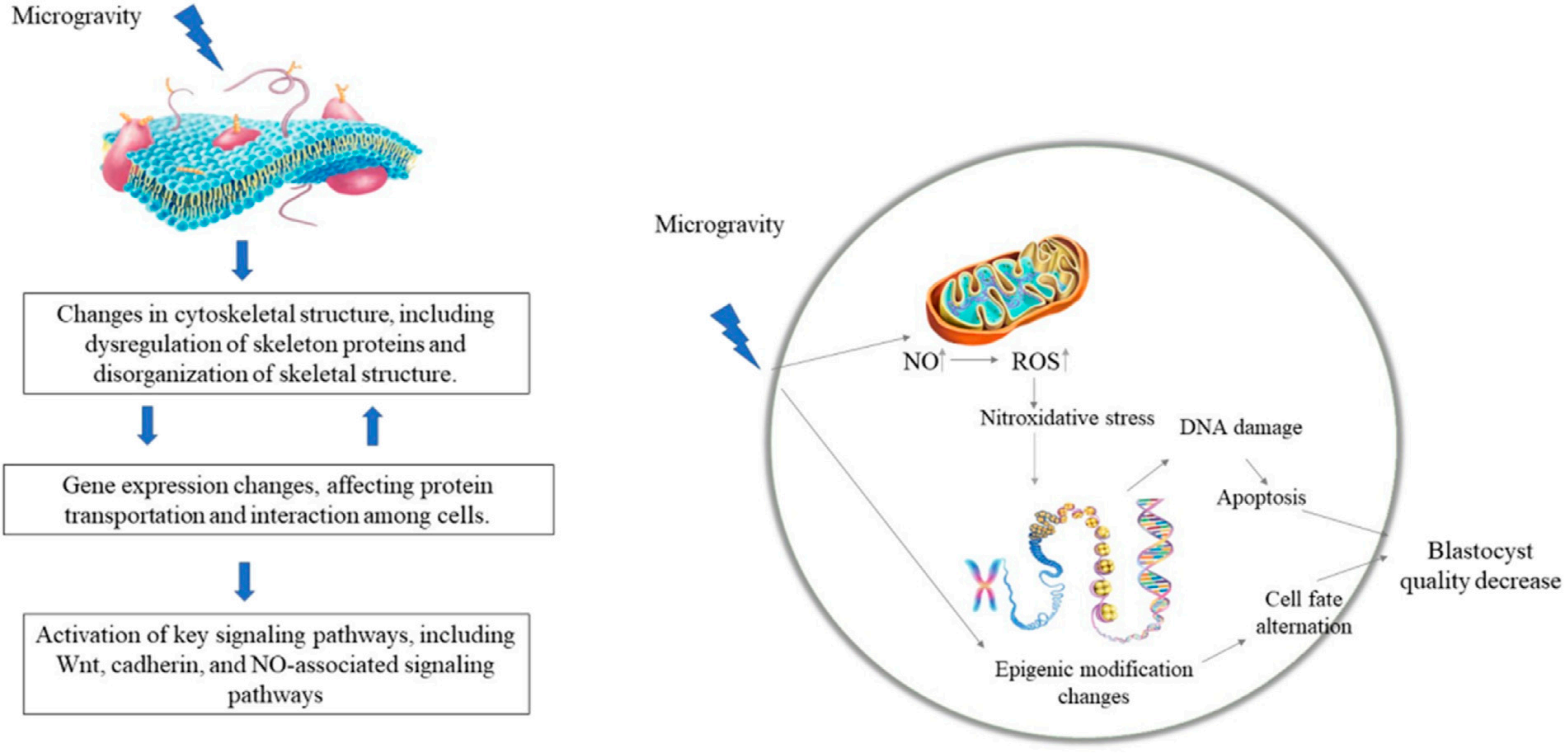
Potential mechanism of microgravity on ESC differentiation and embryonic development. Microgravity may change the cytoskeleton structure and the activity of signaling pathways (such as WNT, NO-related pathways, etc.), thereby regulating the expression of target genes at the transcriptional and epigenetic levels. (Lei et al., 2014; Klein-Nulend et al., 2003; Xiong et al., 2003; Cao et al., 2007; Lei et al., 2020).

Recent research by Lei et al. (2020) suggests that exposure to the space environment can reduce blastocyst development efficiency as well as blastocyst quality and can cause severe DNA damage and hypomethylation of blastocyst cells. Functional clustering analysis of those sites with changed DNA methylation indicate they are associated with histone modification, chromatin and cytoskeletal structure, and RNA metabolism (Lei et al., 2020). Thus, microgravity may participate in early embryonic development by regulating the epigenetic modification of genes and chromatin structure. In summary, microgravity appears to affect embryonic development by inducing the stress response, NO levels, and epigenetic modification (Figure 2), which lack systematic study.

## Future Prospects

Studying the effects and underlying mechanisms of microgravity on ESC differentiation and early embryonic development will help us better understand the impact of space on human reproduction and development and provide a foundation for our exploration and settlement of deep space. Previous studies have shown that microgravity can affect the differentiation of ESCs into the three germ layers and their cells. Optimizing the differentiation of ESCs into various types of cells and organoids under a microgravity environment could help promote their clinical application. Microgravity exposure affects early embryonic development of animals, including mice. Studies on the effects of microgravity on embryonic development will help lay a foundation for the long-term survival and reproduction of humans in outer space. However, due to various technical and experimental limitations, microgravity-related research on ESCs and early embryonic development is still in its infancy. Several exploratory mechanistic studies have shown that signaling pathways, transcription factors, stress response, and epigenetics are involved in the influence of microgravity on ESC differentiation and early embryonic development (Figure 2). However, comprehensive research is still required to clarify the coordinated regulation of these factors on the effects of microgravity.

It should be noted that current simulation devices can only partially simulate the effects of space microgravity. Therefore, the influence of hydrostatic pressure and fluid shear should be considered during microgravity simulation. In addition, as the space environment includes microgravity and radiation, their combined impact should also be considered. Due to the scarcity and high cost of space-based research opportunities, ground-based simulation equipment is an important tool for studying the impact of microgravity. Nevertheless, the results achieved with simulation devices need to be carefully interpreted and verified in space in the future.
